# Impact of heavy proteinuria on clinical outcomes in patients on incident peritoneal dialysis

**DOI:** 10.1186/1471-2369-13-171

**Published:** 2012-12-17

**Authors:** Seok Hui Kang, Kyu Hyang Cho, Jong Won Park, Kyung Woo Yoon, Jun Young Do

**Affiliations:** 1 Department of Internal Medicine, Division of Nephrology, Yeungnam University Hospital, 317-1 Daemyung-Dong, Nam-Ku 705-717, Daegu, Korea

**Keywords:** Peritoneal dialysis, Proteinuria, Residual renal function, Nutrition

## Abstract

**Background:**

There are few reports on the nutritional status changes and residual renal function (RRF) according to proteinuria levels in patients on peritoneal dialysis (PD).

**Methods:**

A total of 388 patients on PD were enrolled. The patients were divided into 3 groups with respect to initial proteinuria: the A (n = 119; <500 mg/day), B (n = 218; 500–3,500 mg/day), and C groups (n = 51; >3,500 mg/day).

**Results:**

The patients with higher proteinuria levels had a higher incidence of male sex, diabetes mellitus, and icodextrin use than those with lower proteinuria levels. Although initial peritoneal albumin loss in C group was lower than that detected in the other groups, no significant difference was observed in peritoneal albumin loss among the 3 groups at the end of follow-up period. At the time of PD initiation, the Geriatric nutritional risk index (GNRI) was lower in the C group than in the other 2 groups. However, at the end of the follow-up period, there was no significant difference in GNRI between the 3 groups. The GNRI increased, and the proteinuria level or RRF decreased more in the C group than in the other 2 groups. There were no significant differences in lean mass index or fat mass index change from the time of PD initiation to the end of the follow-up period. However, fat mass index and nPNA showed greater increases in the C group. The multivariate analysis revealed that proteinuria was negatively correlated with GNRI at the time of PD initiation and at the end of the follow-up period. The initial RRF and proteinuria were negatively correlated with the RRF decline during the follow-up.

**Conclusion:**

The attenuation of the nephrotic proteinuria, along with the RRF decline, was associated with the improvement of the malnutrition.

## Background

Proteinuria is a widely known marker for glomerular/tubulointerstitial disease, with increasing amounts of urinary protein reflecting a greater intensity of kidney injury [1]. Protein losses in the urine are usually compensated for by increased albumin synthesis in patients on peritoneal dialysis (PD) [2]. If protein loss exceeds protein synthesis, malnutrition develops. In addition, proteinuria has been shown to play direct and indirect roles in the deterioration of renal function [1].

Residual renal function (RRF) has consistently been a prognostic factor in patients on PD [3-5]. RRF has been associated with multiple beneficial effects, including blood pressure control, increased sodium concentration or fluid removal, increased middle molecule clearance, and better nutritional status [6]. RRF loss is associated with malnutrition via increased energy expenditure and poorer appetite.

Jansen *et al*. showed that greater urinary protein loss is a risk factor of rapid RRF decline in patients on dialysis [7]. High proteinuria levels in patients on PD are associated with malnutrition and a rapid RRF decline. Over time, patients on PD with high proteinuria levels develop a rapid RRF decline, but their degrees of malnutrition may be improved owing to decreased renal protein loss by the RRF reduction. However, there are few reports on the nutritional status changes and RRF according to proteinuria levels in patients on PD.

## Methods

### Selection of patients

We reviewed the medical records at the Yeungnam University Hospital in Korea and identified 566 patients (>18 years of age) who received PD between January 1997 and May 2011. We excluded 57 patients who had initial oliguria (urine output <200mL/day), 97 patients for whom data on nutritional status or proteinuria levels were unavailable, and 24 patients with less than 1 year of follow-up. A total of 388 patients were enrolled in the study. The patients were divided into 3 groups with respect to initial proteinuria level: the A (n = 119; <500 mg/day), B (n = 218; 500–3,500 mg/day), and C groups (n = 51, >3,500 mg/day). This study was approved by the institutional review board of Yeungnam University Hospital (YUH-12-0366-O35). The board waived the need for informed consent.

### Clinical information

The clinical and laboratory data collected at the time of PD initiation included age, sex, underlying disease of end-stage renal disease (ESRD), use of icodextrin or renin-angiotensin (RAS) blockade, mean arterial pressure (MAP; mmHg), peritoneal equilibration test (PET), serum creatinine level (mg/dL), corrected calcium level (mg/dL), phosphorus level (mg/dL), C-reactive protein level (CRP; mg/dL), serum albumin level (g/dL), 24-hr urine volume (mL/day), RRF (mL/min/1.73m^2^), weekly *Kt/V*, peritoneal *Kt/V*, Geriatric nutritional risk index (GNRI), dialysis modality (automated PD), 24-hr peritoneal albumin loss (mg/day), 24-hr proteinuria level (mg/day), 24-hr dialysate glucose absorption (g/day), lean mass index (kg/m^2^), and fat mass index (kg/m^2^).

The laboratory findings were measured every 6 months after the initiation of PD during the first year. The presence of peritonitis was documented from patient records during the first year. All patients with peritonitis were treated with cefazolin sodium and tobramycin sulfate as first-line antibiotics. For PET, the intra-abdominal fluid was drained, and PD fluid containing 4.25% glucose was infused intraperitoneally [8]. The creatinine level of the drained dialysate 4 hours after the injection was divided by that of blood to obtain the D/P Cr ratio. High transporter was defined as more than 0.81 of D/P Cr ratio. Weekly *Kt/V* was calculated using a 24-hr collection of dialysate and urine. The distribution of volume of urea was calculated with V, estimated from the previous study [9]. Inadequate dialysis was defined as weekly *Kt/V*<1.7. Dietary protein intake was calculated using the following equation: protein equivalent of nitrogen appearance (PNA) = 15.1 + 0.195 urea appearance (mmol/day) + protein losses (g/day) [10]. Urea appearance rate and protein losses were determined from the measured urea and protein excretion in the dialysate and urine. PNA was normalized for ideal body weight calculated using Lorenz equations [11]. Corrected calcium level was calculated on the basis of Payne’s formula as follows: corrected calcium (mg/dL) = total calcium (mg/dL) + 0.8 × [4 – albumin (g/dL)] [12]. Peritoneal albumin loss (mg/day), proteinuria level (mg/day), urine volume, and RRF were measured via the 24-hr urine and dialysate collection. The RRF was calculated from the 24-hr urine sample collection as the mean of the renal creatinine and urea clearances. The GNRI was calculated based on the serum albumin level and body weight, using the following equation: GNRI = [1.489 × albumin (g/L)] + [41.7 × (body weight / ideal body weight)] [13,14]. The ideal body weight was calculated using the Lorentz equations [11]. The ratio of body weight to the ideal body weight was set at 1 when the body weight exceeded the ideal body weight [13,15]. Lean and fat masses were measured using dual X-ray absorptiometry (Hologic, Bedford, MA, USA). The fat and lean mass indexes were calculated by dividing the mass by the height squared. MAP was calculated using the following formula: (2 × systolic blood pressure + diastolic pressure)/3.

### Statistical methods

The data were analyzed using SPSS version 19 (SPSS, Chicago, IL, USA). The distribution of the continuous variables was checked using the Kolmogorov-Smirnov test. The normally distributed variables were expressed as mean (SD) and compared using a *t* test or one-way analysis of variance (ANOVA). The nonparametric variables were expressed as median (range) and were compared using the Mann–Whitney or Kruskal-Wallis test. One-way ANOVA or Kruskal-Wallis test were followed by a post hoc Tukey comparison or Bonferroni correction. The categorical variables were expressed as counts and percentages and analyzed using the Pearson χ^2^ or Fisher exact test. Correlation analysis was performed to assess the strength of the relationship between two continuous variables. Univariate followed by multivariate linear regression was used to determine the independent predictors of the RRF decline. Values of *P* < 0.05 were considered statistically significant.

## Results

### Clinical characteristics at the time of PD initiation and the end of the follow-up period

The mean patient age was 53.3 ± 14.5 years in the A group, 53.3 ± 13.3 years in the B group, and 50.3 ± 12.6 years in the C group (*P* = 0.323; Table 1). The patients with the higher proteinuria levels had a higher incidence of male sex, diabetes mellitus (DM), and icodextrin use than those with lower proteinuria levels. At the time of PD initiation, the GNRI in the C group was lower than that in the other 2 groups. However, at the end of the follow-up period, there was no significant difference in GNRI among the 3 groups. The increases in GNRI and decreases in proteinuria levels or RRF in the C group were greater than those in the other 2 groups (*P* < 0.001; Table 2). At the time of PD initiation, the lean mass index in the C group was greater than that in the other 2 groups (*P* < 0.001). There was no significant difference in lean mass index change from the time of PD initiation to the end of the follow-up period. At the end of the follow-up period, weekly *Kt/V* in the C group was lower than that in the A group (*P* = 0.026). However, there was no significant difference in peritoneal *Kt/V* at either the time of PD initiation or the end of the follow-up period. Although the peritoneal albumin loss was less in the C group than in the other groups at the time of PD initiation, no significant differences were observed in the peritoneal albumin loss at the end of follow-up period. There was no significant difference in change from the time of PD initiation to the end of the follow-up period. 

**Table 1:** Comparison of the clinical characteristics according to the initial proteinuria level at the time of peritoneal dialysis (PD) initiation and the end of the follow-up period

**At the time of PD initiation**	**A group (n=119)**	**B group (n=218)**	**C group (n=51)**	*P *value*
Age (years)	53.3 ± 14.5	53.3 ± 13.3	50.3 ± 12.6	0.323
Sex (male)	55 (46.2%)	123 (56.4%)	36 (70.6%)	0.012
Underlying disease				<0.001
DM	34 (28.6%)	120 (55.0%)	39 (76.5%)	
Hypertension	45 (37.8%)	49 (22.5%)	4 (7.8%)	
Chronic glomerulonephritis	9 (7.5%)	18 (8.3%)	7 (13.7%)	
Others	12 (10.1%)	13 (5.9%)	1 (2.0%)	
Unknown	19 (16.0%)	18 (8.3%)	0	
Use of icodextrin	28 (23.5%)	72 (33.0%)	24 (47.1%)	0.009
Use of renin-angiotensin blockade	101 (84.9%)	171 (78.4%)	46 (90.2%)	0.089
Creatinine level (mg/dL)	6.78 ± 2.54	6.80 ± 2.38	6.94 ± 2.88	0.923
Corrected calcium level (mg/dL)	9.05 ± 0.87	8.88 ± 0.79	8.93 ± 0.84	0.201
Phosphorus level (mg/dL)	3.85 ± 1.28	3.79 ± 1.16	4.05 ± 1.29	0.364
CRP level (mg/dL)	0.177 (0.00~4.52)	0.13 (0.00~4.44)	0.13 (0.02~3.28)	0.186
Dialysis modality (APD)	2 (1.7%)	29 (13.3%)	6 (11.8%)	0.002
PET				0.669
High transporter	17 (14.3%)	30 (13.8%)	7 (13.7%)	
Others	99 (83.2%)	186 (85.3%)	44 (86.3%)	
NA	3 (2.5%)	2 (0.9%)	0 (0%)	
Weekly *Kt/V*	2.44 ± 0.90	2.39 ± 0.63	2.50 ± 0.69	0.604
Peritoneal *Kt/V*	1.75 ± 0.55	1.63 ± 0.47	1.64 ± 0.55	0.096
Inadequate dialysis dose	18 (15.1%)	27 (12.4%)	4 (7.8%)	0.677
Albumin level (g/dL)	3.59 ± 0.48^b^	3.53 ± 0.54^b^	3.05 ± 0.61^a^	<0.001
GNRI	93.3 ± 7.7^b^	93.1 ± 8.42^b^	86.4 ± 9.2^a^	<0.001
nPNA (g/kg/day)	0.88 ± 0.20	0.89 ± 0.18	0.90 ± 0.22	0.900
MAP	90 ± 13	92 ± 12	98 ± 12	0.080
Lean mass index (kg/m^2^)	15.92 ± 2.19^a^	16.53 ± 1.99^a^	17.91 ± 2.64^b^	<0.001
Fat mass index (kg/m^2^)	5.23 ± 2.30	4.91 ± 2.26	4.78 ± 2.34	0.412
Urine volume (mL/day)	700 (200–2830)^a^	960 (200–3300)^b^	1100 (300–3700)^b^	<0.001
RRF (mL/min/1.73 m^2^)	2.90 (0.33–34.30)^a^	3.49 (0.58–15.46) ^a^	4.22 (1.50–23.41)^b^	<0.001
Peritoneal albumin loss (mg/day)	3479 ± 1319^a^	3218 ± 1273^a^	2685 ± 993^b^	0.001
Proteinuria level (mg/day)	150 (39–490)^a^	1241 (504–3,458)^b^	5786 (3,510–24,000)^c^	<0.001
Glucose absorption (g/day)	67.5 ± 19.2	65.4 ± 21.9	65.4 ± 24.8	0.676
At the end of follow-up				
Creatinine (mg/dL)	7.80 ± 2.63^a^	8.50 ± 2.96^a^	10.43 ± 3.35^b^	<0.001
CRP (mg/dL)	0.12 (0.00–3.98)	0.10 (0.00–5.82)	0.15 (0.00–3.75)	0.521
Weekly *Kt/V*	2.33 ± 0.69^b^	2.20 ± 0.64^a,b^	2.01 ± 0.50^a^	0.026
Peritoneal *Kt/V*	1.62 ± 0.39	1.55 ± 0.39	1.60 ± 0.39	0.326
Inadequate dialysis dose	7 (5.9%)	31 (14.2%)	10 (19.6%)	0.058
Albumin (g/dL)	3.80 ± 0.47^b^	3.63 ± 0.47^a^	3.59 ± 0.48^a^	0.008
GNRI	97.2 ± 7.2	94.8 ± 7.5	94.9 ± 7.3	0.122
nPNA (g/kg/day)	0.92 ± 0.19	0.94 ± 0.22	0.96 ± 0.19	0.641
MAP	95 ± 15^a^	96 ± 13^a^	107 ± 15^b^	0.006
Peritonitis rate (episodes/year)	0.32 ± 0.58	0.34 ± 0.58	0.27 ± 0.45	0.720
Lean mass index (kg/m^2^)	15.95 ± 2.32^a^	16.58 ± 2.14^a^	18.03 ± 2.10^b^	<0.001
Fat mass index (kg/m^2^)	5.73 ± 2.32	5.58 ± 2.01	5.41 ± 2.30	0.770
Urine volume (mL/day)	900 (0–2480)^b^	810 (0–2950)^b^	460 (0–1600)^a^	0.001
RRF (mL/min/1.73 m^2^)	3.11 (0.00–24.3)^b^	2.88 (0.00–17.26)^b^	1.41 (0.00–6.14)^a^	0.008
Peritoneal albumin loss (mg/day)	3028 ± 1057	3018 ± 1200	3174 ± 1277	0.606
Proteinuria level (mg/day)	258 (0–4000)^a^	636 (0–4911)^b^	995 (0–15600)^b^	<0.001

The data are expressed as numbers (percentages) for the categorical variables and median (range) or mean ± standard deviation for the continuous variables.

*Statistical significance was tested by one-way analysis of variance or Kruskal-Wallis test for the continuous variables and Pearson χ^2^ test or Fisher exact test for the categorical variables. The different superscripts (^a,b,c^) indicate significant differences from each other using post hoc Tukey comparison or Bonferroni correction.

DM, diabetes mellitus; CRP, C-reactive protein; APD, automated peritoneal dialysis; PET, peritoneal equilibration test; NA, non-available; GNRI, geriatric nutritional risk index; nPNA, normalized protein equivalent of nitrogen appearance; MAP, mean arterial pressure; RRF, residual renal function.

**Table 2:** Changes in the variables from the time of peritoneal dialysis initiation to the end of follow-up period: comparison of the 3 groups according to initial proteinuria levels

	**A group**	**B group**	**C group**	*P *value*
Delta GNRI	3.47 ± 6.54^a^	1.92 ± 6.63^a^	6.97 ± 8.52^b^	<0.001
Delta RRF (mL/min/1.73 m^2^)	0.00 (−9.99 to 7.10)^a^	−0.81 (−7.88 to 6.58)^a^	−2.41 (−23.41 to 2.31)^b^	<0.001
Delta nPNA	0.04 ± 0.23	0.05 ± 0.23	0.06 ± 0.24	0.898
Delta MAP	4.4 ± 16.3	3.2 ± 17.1	8.6 ± 15.4	0.483
Delta total lean mass index	0.47 ± 1.19	0.10 ± 1.23	−0.07 ± 2.56	0.198
Delta total fat mass index	0.76 ± 1.03	0.75 ± 1.87	1.20 ± 1.49	0.418
Delta peritoneal albumin loss (mg/day)	−233 ± 995	−59 ± 1370	324 ± 1477	0.075
Delta proteinuria (mg/day)	197 ± 648^a^	−475 ± 945^a^	−4626 ± 5466^b^	<0.001

The data are expressed as numbers (percentages) for the categorical variables and median (range) or mean ± standard deviation for the continuous variables.

*Statistical significance was tested by one-way analysis of variance or Kruskal-Wallis test for the continuous variables and the Pearson χ^2^ test or Fisher exact test for the categorical variables. The different superscripts (^a,b,c^) indicate significant differences from each other using post hoc Tukey comparison or Bonferroni correction.

GNRI, geriatric nutritional risk index; RRF, residual renal function; nPNA, normalized protein equivalent of nitrogen appearance; MAP, mean arterial pressure.

### Factors affecting GNRI and RRF decline

To identify the relevance of the variables affecting GNRI and RRF decline, clinical and laboratory parameters were analyzed using univariate and multivariate regression models (Table 3 and Table 4). The univariate and multivariate analyses revealed that age, DM, and proteinuria were negatively correlated with GNRI at the time of PD initiation. At the end of the follow-up period, age, DM, peritoneal albumin loss, proteinuria, and RRF were associated with GNRI. The initial RRF and proteinuria levels were negatively correlated with RRF decline during the follow-up. Notably, there was no significant association between RRF decline and MAP at PD initiation. 

**Table 3:** Predictive factors affecting the Geriatric nutritional risk index at the time of peritoneal dialysis (PD) initiation and the end of the follow-up period

**At the time of PD initiation**	**Univariate**		**Multivariate**	
	**Standardized β ± SE**	***P *value**	**Standardized β ± SE**	***P *value**
Age (years)	−0.142 ± 0.033	0.006	−0.115 ± 0.032	0.022
Use of icodextrin	0.031 ± 0.941	0.552	-	-
Sex (female)	0.014 ± 0.889	0.782	-	-
DM	−0.265 ± 0.852	<0.001	−0.185 ± 0.882	<0.001
Peritoneal albumin loss (mg/day)	−0.021 ± 0.000	0.683	-	-
RRF (mL/min/1.73 m^2^)	0.051 ± 0.150	0.327	-	-
Proteinuria level (mg/day)	−0.290 ± 0.000	<0.001	−0.250 ± 0.000	<0.001
At the end of follow-up				
Age (years)	−0.176 ± 0.032	0.003	−0.208 ± 0.031	<0.001
Use of icodextrin	0.062 ± 0.906	0.291	-	-
Sex (female)	0.007 ± 0.880	0.899	-	-
DM	−0.291 ± 0.839	<0.001	−0.177 ± 0.864	0.003
Peritoneal albumin loss (mg/day)*	−0.207 ± 0.000	<0.001	−0.204 ± 0.000	<0.001
RRF (mL/min/1.73 m^2^)*	0.189 ± 0.151	0.001	0.212 ± 0.145	<0.001
Proteinuria level (mg/day)*	−0.143 ± 0.000	0.016	−0.143 ± 0.000	0.014

*The values are presented as findings at the end of the follow-up.

SE, standard error; DM, diabetes mellitus; RRF, residual renal function.

**Table 4:** Predictive factors affecting the decline in residual renal function (RRF) from the time of peritoneal dialysis initiation to the end of the follow-up period

	**Univariate**		**Multivariate**	
	**Standardized β ± SE**	***P *value**	**Standardized β ± SE**	***P *value**
Age	0.008 ± 0.012	0.897	-	-
Sex (female)	0.122 ± 0.321	0.038	−0.064 ± 0.278	0.211
Use of icodextrin	−0.092 ± 0.333	0.118	-	-
DM	−0.096 ± 0.321	0.103	-	-
MAP (mmHg)*	0.087 ± 0.018	0.242		
RRF (mL/min/1.73m^2^)*	−0.517 ± 0.045	<0.001	−0.505 ± 0.045	<0.001
Peritoneal albumin loss (mg/day)*	−0.117 ± 0.000	0.050	-	-
Proteinuria level (mg/day)*	−0.315 ± 0.000	<0.001	−0.271 ± 0.000	<0.001

* The values are presented as the findings at the time of peritoneal dialysis initiation.

MAP, mean arterial pressure; DM, diabetes mellitus; SE, standard error.

## Discussion

This study demonstrates that a high proteinuria level is associated with malnutrition and rapid RRF or urine volume decline. A simultaneous decline in proteinuria level and RRF or urine volume appears to improve initial low GNRI. However, the RRF and urine volume decline led to decreased dialysis adequacy.

Proteinuria is a well-documented risk factor for the progression of chronic kidney disease in patients who are not treated with dialysis [16]. However, the specific effects of proteinuria level on RRF in patients on dialysis remain elusive. Jansen *et al*. showed that greater urinary protein loss is negatively associated with RRF [7]. However, using multivariate analysis, another study did not show that 24-hr proteinuria is associated with a faster RRF decline [17]. This study demonstrates that high proteinuria levels in patients on PD are associated with a rapid RRF decline. In this study, the RRF decline was 0.00 (−9.99 to 7.10) mL/min/1.73 m^2^ in the A group and −0.81 (−7.88 to 6.58) mL/min/1.73 m^2^ in the B group; in contrast, it was −2.41 (−23.41 to 2.31) mL/min/1.73 m^2^ in the C group. Although RRF decline occurred in the B and C groups, the RRF in the patients with high proteinuria levels tended to show a steeper decline. The multivariate regression analysis showed similar results.

Malnutrition is associated with mortality and morbidity in patients on dialysis [18]. Hypoalbuminemia or protein energy wasting in PD patients is related to increased catabolism, decreased protein synthesis, and external protein loss from peritoneum or urine [18-20]. External protein loss can have an identical pathogenic effect on nephrotic syndrome [20]. The loss of peritoneal protein in PD patients averages 5 g/day [21,22]. One study demonstrated that peritoneal protein loss is related to mortality and malnutrition in patients on PD [20]. However, protein losses in the peritoneal effluent are usually compensated for by increased protein synthesis [23]. Balafa *et al*. showed that baseline peritoneal albumin and protein clearances are associated with comorbidities, but this is not a risk factor for decreased survival per se [22]. In the present study, the peritoneal albumin loss was less in the C group than in the A group at the time of PD initiation. High proteinuria levels are associated with hypoalbuminemia, which may be associated with decrease in peritoneal albumin loss. However, no significant difference was observed in peritoneal albumin loss among the 3 groups at the end of follow-up period. Compared with changes in proteinuria, those in peritoneal albumin loss were small (delta peritoneal albumin loss was −233 ± 995 mg/day in A group, –59 ± 1370 in B group, and 324 ± 1477 mg/day in C group). These data demonstrate the lack of a relationship between proteinuria and peritoneal albumin loss. In linear regression, the peritoneal albumin loss was negatively correlated with the nutritional status at the end of the follow-up period. Our data show that the initial nutritional status was associated with proteinuria alone, that follow-up nutritional status was associated with proteinuria and peritoneal albumin loss. High proteinuria levels may have a greater effect on nutritional status than peritoneal albumin loss.

The patients on PD who had high proteinuria levels showed rapid RRF decline and eventual improvement of nutritional status. The GNRI scores increased from 86.4 ± 9.2 to 94.9 ± 7.3 in the C group. The C group demonstrated a significantly higher initial protein loss in the urine accompanied by malnutrition. However, the follow-up GNRI was not significantly different between the C group and the other 2 groups. The impact of the initial high proteinuria level on malnutrition was attenuated, along with a decreased RRF with increasing duration of PD. The change in the serum albumin level was similar to that in GNRI.

Preservation of RRF has been consistent independent predictor of survival in patients on PD [3-5,24]. The benefits include improved control of blood pressure and anemia, removal of uremic toxin, superior phosphate control, and dialysis adequacy [6]. Wang *et al*. showed that RRF preservation is associated with better nutritional status according to appetite improvement or the removal of inflammatory mediators [24]. However, uncontrolled high proteinuria levels are related to malnutrition. In fact, patients with uncontrolled high proteinuria levels who are not on dialysis could be treated with medical nephrectomy using iatrogenic nephrotoxic drugs. This study shows that RRF combined with heavy proteinuria is associated with malnutrition and that a rapid RRF decline leads to improved nutritional status. This study also shows that RRF declines lead to decreased dialysis adequacy. Because there were no significant differences in peritoneal *Kt/V* between the 3 groups at baseline or follow-up, the decline of the weekly *Kt/V* may be associated with the decreased renal clearance. However, there was no significant difference in number of patients with inadequate dialysis.

Previous studies have shown that RRF loss is associated with malnutrition in patients on PD [24-26]. However, these studies have limitations that differ from those of the present study. First, they had a cross-sectional design. Anuria or low RRF as a baseline characteristic was associated with poor nutritional status, but there are few data on the effects of RRF decline on nutrition. Second, patients were divided into groups according to RRF status. Patients with heavy proteinuria may be enrolled into groups along with patients with RRF. If patients with RRF were divided into groups with respect to proteinuria, subgroup analyses may show similar results. The present study had an observational design and simply monitored changes in RRF status. Patients with RRF were divided into 3 groups according to proteinuria status.

The patients with nephrotic syndrome and normal renal function have increased protein synthesis rates but inadequate responses to normalized serum albumin levels [27]. Patients on dialysis also experience increased protein synthesis in response to protein loss, but the response to protein loss in patients on dialysis is greater than that in patients with nephrotic syndrome and normal renal function [2,28]. Therefore, this malnutrition change may be more closely associated with increases in protein catabolic rate than renal function. The present study did not show whether the nutritional improvement in patients on PD with nephrotic-range proteinuria levels is associated with changes in protein catabolic rate or decreases in proteinuria levels. Further investigations are needed to determine the definite association between improved nutrition and RRF decline or decreased proteinuria levels.

In the present study, MAP was not associated with a decline in RRF, and at the end of the follow-up period, MAP in C group was higher than that in the other groups. Previous studies have shown that MAP in the post-dialysis period is not associated with a decline in RRF in patients on PD [29,30]. However, diastolic blood pressure at predialysis period is related to decline in RRF [7]. The relation between blood pressure and RRF remains controversial. The higher MAP observed in C group at the end of follow-up period may be associated with rapid decline in RRF or underlying disease (high incidence of DM in the C group). In our cohort, MAP at the end of the follow-up period was 102 ± 14 mmHg in patients with RRF < 1 mL/min/1.73m^2^ and 97 ± 12 mmHg in those with RRF ≥ 1 mL/min/1.73m^2^ (*P* = 0.020). The RAS blockade is a well-known therapeutic target associated with RRF preservation [6]. Most patients in the present study received a RAS blockade, and we were unable to analyze the association between RAS blockade use and RRF decline.

It is important to evaluate whether aminoglycoside use is associated with RRF decline in patients on PD. Some previous studies have shown variable results regarding the association between aminoglycoside use and rapid RRF decline [17,31,32]. A multicenter study recently revealed that empiric treatment with aminoglycoside for peritonitis was not associated with a rapid RRF decline [33]. In present study, all patients with peritonitis received aminoglycoside as first-line antibiotics. There was no significant difference in peritonitis rate or aminoglycoside use among the 3 groups. Investigations that enroll only patients with peritonitis are needed to determine the effect of aminoglycoside on RRF decline.

Serum albumin is not the gold standard of nutrition in patients on PD [34]. Serum albumin is greatly influenced by variable factors such as malnutrition, hydration status, and inflammation. This variable is included in the GNRI equation. Therefore, it is difficult to consider GNRI as a reliable measure of nutrition in patients with heavy proteinuria levels. At the time of PD initiation, the differences in GNRI among the 3 groups may be not associated with nutritional status. The present study shows data for nPNA, lean mass index, and fat mass index using DEXA. The lean mass index in the C group was greater than that in the other 2 groups at the time of PD initiation and the end of the follow-up period. However, there was no significant difference in lean mass index change from the time of PD initiation to the end of the follow-up period. However, because overhydration causes overestimation of lean mass by DEXA, the initial lean mass levels in C group may have been overestimated. Therefore, the increases in lean mass in C group may be higher than those in the other groups. There was no significant difference in fat mass index among the 3 groups, but we did detect a greater increase in fat mass index in the C group (delta fat mass index: of 0.76 ± 1.03, 0.75 ± 1.87, and 1.20 ± 1.49 in the A, B, and C groups, respectively). The same trend was seen in nPNA.

## Conclusion

The high proteinruia level at PD initiation was associated with rapid RRF decline. The attenuation of the nephrotic proteinuria, along with the RRF decline, was associated with the improvement of the malnutrition.

## Competing interests

No competing interests to declare.

## Authors’ contributions

SHK designed research, performed, and wrote the paper. JWP and KHC interpreted the data. KYW contributed to acquisition of data. JYD was involved in revising the manuscript critically for important intellectual content and have given final approval of the version to be published. All authors read and approved the final manuscript.

## Pre-publication history

The pre-publication history for this paper can be accessed here:

http://www.biomedcentral.com/1471-2369/13/171/prepub
